# Influence of factor XIII activity on post-operative transfusion in congenital cardiac surgery—A retrospective analysis

**DOI:** 10.1371/journal.pone.0199240

**Published:** 2018-07-10

**Authors:** Fabian B. Fahlbusch, Thomas Heinlein, Manfred Rauh, Sven Dittrich, Robert Cesnjevar, Julia Moosmann, Jennifer Nadal, Matthias Schmid, Frank Muench, Michael Schroth, Wolfgang Rascher, Hans-Georg Topf

**Affiliations:** 1 Department of Pediatrics and Adolescent Medicine, Friedrich-Alexander-University of Erlangen-Nürnberg, Erlangen, Germany; 2 Department of Pediatric Cardiology, Friedrich-Alexander-University of Erlangen-Nürnberg, Erlangen, Germany; 3 Department of Pediatric Cardiac Surgery, Friedrich-Alexander-University of Erlangen-Nürnberg, Erlangen, Germany; 4 Institute of Medical Biometry, Informatics and Epidemiology (IMBIE), University Hospital, Bonn, Germany; 5 Cnopf’sche Kinderklinik, Diakonie Neuendettelsau, Nürnberg, Germany; University of Bern, University Hospital Bern, SWITZERLAND

## Abstract

**Objectives:**

Coagulation factor XIII (FXIII) plays a key role in fibrin clot stabilization—an essential process for wound healing following cardiothoracic surgery. However, FXIII deficiency as a risk for post-operative bleeding in pediatric cardiac surgery involving cardiopulmonary bypass (CPB) for congenital heart disease (CHD) is controversially discussed. Thus, as primary outcome measures, we analyzed the association of pre-operative FXIII activity and post-operative chest tube drainage (CTD) loss with transfusion requirements post-operatively. Secondary outcomes included the influence of cyanosis and sex on transfusion.

**Methods:**

Our retrospective analysis (2009–2010) encompassed a single center series of 76 cardio-surgical cases with CPB (0–17 years, mean age 5.61 years) that were post-operatively admitted to our pediatric intensive care unit (PICU). The observational period was 48 hours after cardiac surgery. Blood cell counts and coagulation status, including FXIII activity were routinely performed pre- and post-operatively. The administered amount of blood products and volume expanders was recorded electronically, along with the amount of CTD loss. Uni- and multivariate logistic regression analysis was performed to calculate the associations (odds ratios) of variables with post-operative transfusion needs.

**Results:**

FXIII activities remained stable following CPB surgery. There was no association of pre- and post-operative FXIII activities and transfusion of blood products or volume expanders in the first 48 hours after surgery. Similarly, FXIII showed no association with CTD loss. Cyanosis and female sex were associated with transfusion rates.

**Conclusions:**

Although essentially involved in wound healing and clotting after surgery, FXIII activity does not serve as a valid predictor of post-operative transfusion need.

## Introduction

**Fig 1:**
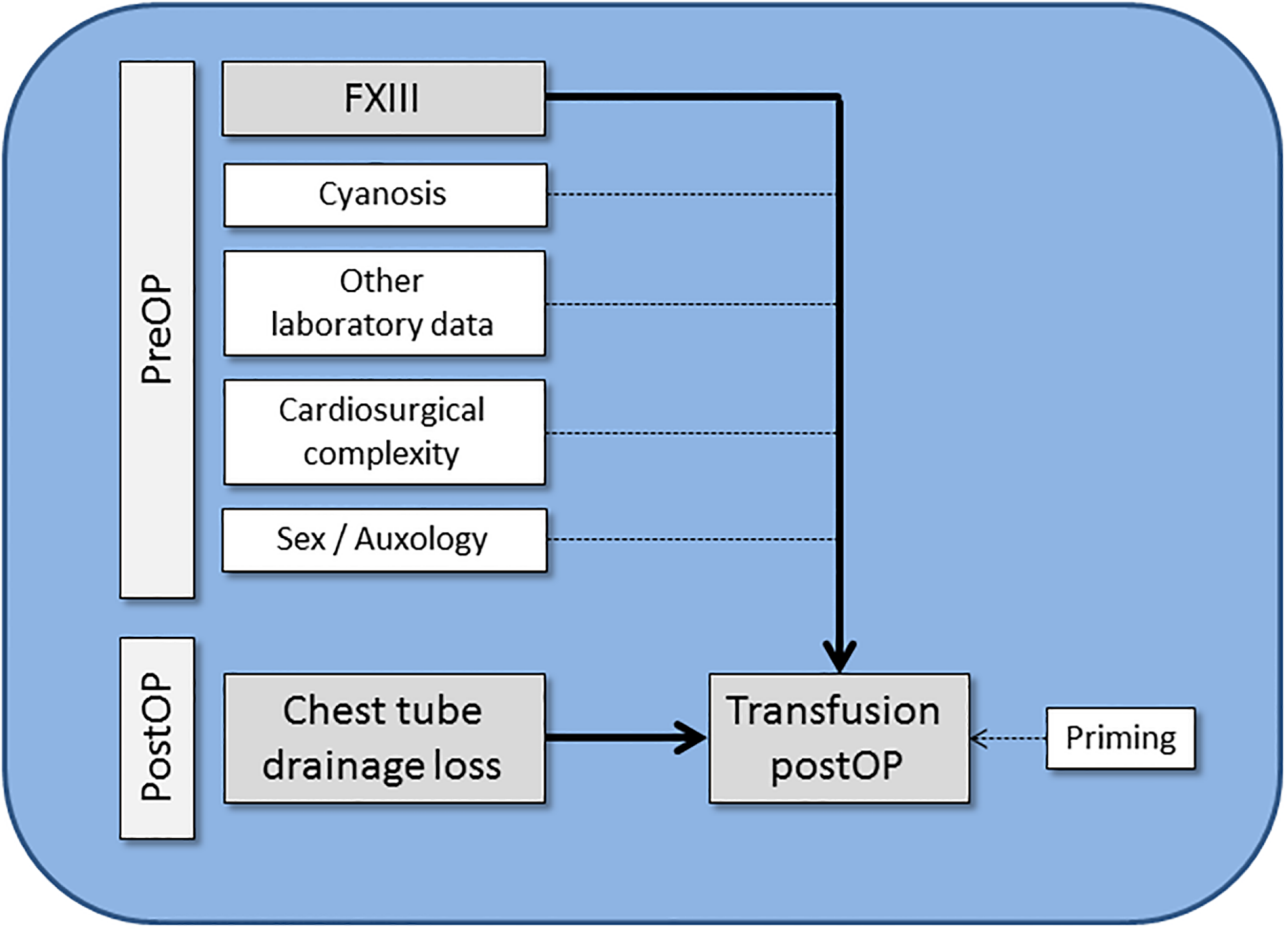
Overview of the primary (large font, grey background) and secondary measures (small font, white background) of our study. Abbreviations: preOP: pre-operatively, postOP: post-operatively, FXIII: factor XIII.

Activation of the coagulation cascade following (surgical) tissue injury is essential for the prevention of blood loss and for the initiation of wound healing. Coagulation factor (F) XIII, plays a key role in fibrin clot stabilization and cross-linking of anti-fibrinolytic proteins to the clot [1]. Recent findings (reviewed by [1]), recognize the transglutaminase FXIII as a multifunctional protein involved in regulatory mechanisms, as well as construction and repair processes beyond hemostasis (e.g. maintenance of pregnancy). Wound healing following cardiothoracic operations especially requires the formation of a stable fibrin surface on injured tissue to avoid mechanical disruption, e.g. by thoracic cage movement and heart or lung action. Loss of FXIII activity results in premature breakdown of otherwise intact fibrin clots, which in turn delays the process of wound healing by recurrent bleeding [2]. Peri-operative acquired factor XIII deficiency is considered to be a potential risk factor for post-operative bleeding following “open heart” surgery with cardiopulmonary bypass (CPB) [3–5] or surgical interventions involving the coronary arteries [6, 7]. Additionally, the risk for post-operative bleeding is increased by the use of volume expansion for CPB with consecutive dilutional coagulopathy [8], which involves loss, consumption, or dilution of coagulation factors. Iatrogenic blood replacement with fluids lacking adequate coagulation factors [9] plays a central role and defines the dynamics of dilutional coagulopathy [10]. Furthermore, hemostasis following surgery might be aggravated by hypothermia, acidosis and fibrinolysis, thus resulting in a worsening of patient’s outcome [11]. Gertler et al. [12] on the other hand, found that FXIII activity in infants with congenital heart defects (CHD) was within the lower range of normal adults and independent of the presence of cyanosis and the patient’s age. The peri-operative role of FXIII in hemostasis and chest tube drainage (CTD) loss in children is controversially discussed. In a previous study, we found that treatment with plasmatic FXIII significantly reduced severe pleural effusion in the first 24 hours (h) following “open-heart” surgery [13] in children beyond 1 year of age. Transfusion requirements, however, were not studied [13]. In contrast, Gertler at al. [12] showed that in infants (<1 year) with CHD pediatric cardiac surgery had no significant influence on FXIII plasma levels, which in turn did not contribute to increased blood and/or chest tube losses and transfusion requirements post-operatively. Thus, as primary outcome measures, we analyzed the association of pre-operative FXIII activity and post-operative chest tube drainage (CTD) loss with transfusion requirements post-operatively. Secondary outcomes included the influence of cyanosis and sex on transfusion (see Fig 1). Our retrospective analysis encompassed a single center series of 76 cases (age 0–17 years) that were post-operatively admitted to our pediatric intensive care unit (PICU) from July 2009 to April 2010.

## Materials and methods

### Patients and data acquisition

Our investigation conforms to the principles outlined in the Declaration of Helsinki [14]. The local Clinical Ethics Committee at the University Hospital Erlangen provided a waiver of approval for the study (#337_17 Bc). Descriptive patient’s peri-operative characteristics are displayed in Table 1. SDS values for weight were calculated according to Kromeyer-Hauschild et al. [15]. Table 2 lists the leading diagnosis and respective surgical procedures. Our retrospective study (07/2009-04/2010) encompasses a single center series (Department of Pediatric Cardiac Surgery at the Friedrich-Alexander-University of Erlangen-Nürnberg, Germany). During that period open cardiothoracic surgery for CHD was performed in 99 patients. Of those, 23 did not require CPB and were subsequently excluded from our study. Of the remaining 76 cases (all Caucasian, 36 females (47.4%), 40 males (52.6%)) eight were operated under beating heart condition. Open cardiothoracic surgery for CHD on CPB was performed following the institutional protocols described below.

**Table 1:** Descriptive analysis of peri-operative data.

**Pre-operative auxologic data**:		
Total cohort body weight (kg)	11.15	(2.77–73.00)
Total cohort body weight (SDS)	-1.25	(-3.65–3.20)
≥10th Pct. (n/%)	39	(51.32%)
<10th Pct. (n/%)	37	(48.68%)
<3rd Pct. (n/%)	21	(27.63%)
ReOP body weight (SDS)	-1.24	(-2.71–1.76)
≥10th Pct. (n/%)	11	(52.38%)
<10th Pct. (n/%)	10	(47.62%)
<3rd Pct. (n/%)	4	(19.05%)
Age (d)	803.00	(3.0–6126)
Age (years, LMS)	2.29	(0.83–17.083)
Above 3 yoa (n = 36)	47.37%	(3.25–17.083)
Below 3 yoa (n = 40)	52.36%	(2.5–3.0)
Age ReOP (years, LMS)	8.17	(0.17–16.25)
**Pre-operative blood test**:		
Hemoglobin (g/dl)	13.25	(7.5–19.2)
Platelet count (n/μl)	264.0 x 10^3^/μl	(114.0–564.0 x 10^3^/μl)
Prothrombin time ratio (%)	88.9	(46.0–125.0)
FXIII activity (%)	79.00	(42.0–156.0)
**Post-operative blood test**:		
Hemoglobin (g/dl)	11.60	(8.7–17)
Platelet count (n/μl)	202.0 x 10^3^/μl	(96.0–415.0 x 10^3^/μl)
Prothrombin time ratio (%)	73.0	(41.0–141.0)
FXIII activity (%)	78.0	(44.0–122.0)
**Post-operative blood test—percent change (**Δ**) from pre-operative test**:		
ΔHemoglobin (mean ± SD)	0.865 ± 0.168	p<0.0001*
ΔPlatelet count (mean ± SD)	0.809 ± 0.316	p<0.0001*
ΔProthrombin time ratio (mean ± SD)	0.914 ± 0.379	p<0.0001*
ΔFXIII activity (mean ± SD)	1.020 ± 0.286	p<0.039*
**Details of surgical procedure**:		
RACHS-1 category	3.0	(1.0–6.0)
	1	(11.84%)
	2	(31.58%)
	3	(44.74%)
	4	(9.21%)
	5	(0.0%)
	6	(2.63%)
Aristotle Score (max. 15 pts)	7.5	(3.0–14.5)
	3–5	(14.47%)
	6–7.5	(42.11%)
	8–9.5	(25.0%)
	10–14.5	(18.42%)
Duration of CPB (min)	143.00	(46.0–330.0)
Cross clamp time (min)	81.50	(5.0–241.0)

All data are given as median and range, unless otherwise stated.

* Paired Student’s t-test. Abbreviations: CPB = cardiopulmonary bypass, ml = milliliters, kg = kilograms, h = hours, d = days, min = minutes, yoa = years of age, Pct. = percentile, ReOP = re-operation, SDS = standard deviation score, LMS = LMS parameters are the median (M), the generalized coefficient of variation (S), and the power in the Box-Cox transformation (L).

**Table 2:** List of cardiac diagnosis and performed surgical procedure involving CPB.

Diagnosis	Operation	n
ASD (incl. sinus venosus)	Patch closure	10
VSD	Patch closure	11
Aortic arch hypoplasia	Reconstruction of the aortic arch	2
d-TGA	N = 3 atrial switch operation N = 2 pulmonary patch	5
cAVSD	Correction of AVSD	3
RVOT-reconstruction	N = 4 pulmonary valve replacement N = 2 RVOT-patch	6
Single ventricle	N = 1 aorto-pulmonary shunt N = 3 Norwood N = 4 Bidirectional Glenn-anastomosis N = 1 Fontan	9
TAC	N = 2 replacement of pulmonary conduit N = 1 correction with VSD and PFO closure+ conduit implantation	3
Aortic valve disease	Aortic valve replacement	6
pAVSD	N = 2 reconstruction of the atrioventricular valve + ASD closure N = 1 mitral valve reconstruction + pacemaker implantation	3
Fallot-Tetralogy (TOF)	TOF repair	3
Interrupted aortic arch	N = 1 aortic arch repair with patch N = 1 end-to-end anastomosis	2
Pulmonary venous obstruction	N = 2 PAPVC-repair N = 1 TAPVC-repair N = 2 Cor triatriatrum repair	5
Tricuspid valve disease	N = 4 tricuspid valve reconstruction (incl. two Ebstein malformations)	4
Supravalvular aortic stenosis	Patch enlargement of supravalvular aortic stenosis	1
DCM	Aortic root replacement, pacemaker implantation	1
Mitral valve insufficiency	Mitral valve reconstruction, Ring implantation	1
PA/IVS	Aorto-pulmonary shunt	1
**Total**		**76**

Total cohort size was n = 76, with 47.4% female and 52.6% male patients; 72.4% underwent cardiac surgery for the first time, 27.6% were re-operated. In 10.5% of the cases intervention was performed as beating heart surgery. Abbreviations: ASD: atrial septal defect; VSD: ventricular septal defect; d-TGA: dextro transposition of the great arteries; c/pAVSD: complete/partial atrio-ventricular septal defect; RVOT: right ventricular outflow tract; TAC: Truncus arteriosus communis; TOF: Tetralogy of Fallot; P/TAPVC: partial/total anomalous pulmonary venous connection; DCM: dilated cardiomyopathy; PFO: patent foramen ovale; PA/IVS: pulmonary atresia with intact ventricular septum.

We utilized standardized scores (Risk Adjustment for Congenital Heart Surgery/RACHS-1 [16] and Aristotle score [17]) to adjust for baseline risk differences of in-hospital mortality and comprehensive complexity (Table 1) to ensure comparability of cases examined [16]. For RACHS-1 score, a national U.S. panel of pediatric cardiologists and cardiac surgeons stratified surgical procedures and their underlying anatomic diversity into six risk categories using clinical judgement. Taking into account the patient’s age, type of surgery and similar in-hospital mortality, the RACHS-1 method aims at adjusting for baseline risk differences, thereby facilitating comparisons of in-hospital outcome data for groups of children undergoing surgery for CHD [16]. The international Aristotle committee established a score that ranks the complexity of a cardio-surgical case based on the procedure (its potential for mortality, morbidity and the anticipated technical difficulty) and further adjusts for procedure-independent patient characteristics (so-called comprehensive Aristotle score). The complexity of cardio-surgical procedures can thus be determined by score or score-based category (similar to RACHS-1), as follows: level 1 = 1.5–5.9, level 2 = 6.0–7.9, level 3 = 8.0–9.9 and level 4 = 10.0–15.0 [17].

The protocols and the team of surgeons, anaesthetists and paediatric cardiologists remained unchanged during the study period. In 55 cases primary cardiac surgery was performed, while 21 patients underwent their 2^nd^ surgical intervention. The duration of CPB and cross clamp time, as well as intra-operative CPB priming and transfusion requirements (S1 Table) were recorded by the attending cardio-technician and were electronically documented (Soarian Clinicals 4.00 SP10, Cerner Health Services Inc., Idstein, Germany). Retrospective data acquisition was performed using the laboratory information system Lauris (version 15.09.29.9, Swisslab GmbH, Berlin, Germany) and Integrated Care Manager (ICM, Drägerwerk AG & Co. KGaA, Lübeck, Germany) software solutions. Post-operative CTD loss (ml) was continuously monitored and electronically noted (ICM) by the critical care nursing staff. In the following CTD loss within the first 48 h post-operatively is referred to as “CTD loss”. The total amount of post-operatively used allogeneic blood products was recorded starting from the time of admittance to the PICU for 48 h. Table 3 displays post-operative data (chest tube loss, transfusion, duration of stay). Exclusion criteria were pre-operative coagulopathies.

**Table 3:** Descriptive analysis of post-operative data.

**Chest tube loss 48h post-operatively (mean ± SD)**:	
Total (ml)	321.38 ± 368.55
Relative (ml/kg)	29.434 ± 44.69
**Transfusion (mean ± SD)**:	
**PRBC (n = 36)**:	
total (ml)	176.19 ± 148.25
relative (ml/kg)	22.77 ± 22.77
< 15 ml/kg	19 (25.0%)
15–30 ml/kg	9 (11.8%)
30–50 ml/kg	3 (3.9%)
> 50 ml/kg	5 (6.6%)
**Platelets (n = 13)**:	
total (ml)	103.38 ± 47.37
relative (ml/kg)	12.71 ± 9.22
< 15 ml/kg	10 (13.2%)
15–30 ml/kg	2 (2.63%)
> 30 ml/kg	1 (1.3%)
**FFP (n = 42)**:	
total (ml)	231.14 ± 348.05
relative (ml/kg)	31.08 ± 61.61
< 15 ml/kg	22 (28.9%)
15–30 ml/kg	10 (13.2%)
> 30 ml/kg	10 (13.2%)
**HES 6% (130/04, n = 27)**:	
total (ml)	357.59 ± 359.95
relative (ml/kg)	16.87 ± 11.19
< 15 ml/kg	16 (21.1%)
15–30 ml/kg	7 (9.2%)
> 30 ml/kg	4 (5.3%)
Others:	
**PCC / Fibrinogen**	n = 4 / n = 3
**AT III**	n = 4
**Duration of stay (median and range)**:	
Pre- and post-operatively (h)	47.00 (16.0–622.0)
Post-operatively (h)	45.00 (15.0–427.0)

Abbreviations: ATIII = antithrombin III, PRBC = packed red blood cells, FFP = fresh frozen plasma, PCC = prothrombin complex concentrate, HES = hydroxyethyl starch, ml = milliliters, kg = kilograms, h = hours.

### Blood sampling and analysis

Routinely, venous blood was drawn 1–2 days prior to surgery as pre-operative check-up and directly post-operatively on our PICU, collected in tubes (Sarstedt, Nümbrecht, Germany) containing trisodium citrate as anticoagulant (ratio blood / anticoagulant = 9:1) or EDTA. Blood cell counts were measured using a blood cell analyser XE-2100 (Sysmex, Norderstedt, Germany), as previously described [18]. Platelet poor citrate plasma was obtained by centrifugation (10 min, 1 600*g) at room temperature (RT). Supernatant plasma was collected and centrifuged (10 min, 22,000 g, RT) in a micro-centrifuge to minimize residual platelets. Determination of Prothrombin time ratio (PT, % of reference) and FXIII activity (%) was performed in a BCS^®^ analyzer (Siemens, Marburg, Germany), using commercially available reagents [18]. FXIII activity can be indirectly monitored via ammonia release [19] that follows FXIII-catalyzed peptide cross-linkage to glycine ethyl esters [12]. The ammonia release can be enzymatically quantified by monitoring absorbance at 340 nm. Expected FXIII levels in healthy children are age dependent [20], with a range of 42 to >156%. FXIII BCS measurements remain uninfluenced by high levels of heparin observed during the course of CPB [12].

### Institutional protocol for transfusion and factor administration

Intra- and post-operative indication for transfusion was alike and followed our departmental transfusion algorithm: packed red blood cells (PRBC) were administered at a hemoglobin (Hb) level of 14 g/dl in cyanotic patients and 10 g/dl in non-cyanotic patients. In the case of on-going bleeding, fresh frozen plasma (FFP, 10-15ml/kg) was transfused if PT reached values below 50%. Platelets were transfused at a platelet count below 50 x 10^3^/μl. In the case of bleeding, the indication for re-exploration was determined by the attending surgeon. In cases where these first-line measures remained ineffective to stabilize post-operative bleeding, other factors (all from CSL Behring GmbH, Marburg, Germany) were intravenously deployed: Aiming at fibrinogen levels of > 150mg/dl, 1 g fibrinogen concentrate (Haemocomplettan P) per m^2^ body surface area (BSA) was administered, where necessary (n = 3). Prothrombin complex concentrate (PCC, Beriplex P/N, 40IE/kg, 1ml/h) administration was performed (n = 4), if severe bleeding occurred, with antithrombin III (ATIII) >80% as a prerequisite. ATIII (Kybernin, 100 IE/kg/24h continuous i.v.) itself was given to 4 patients. Our study did not involve cases with continuous bleeding despite adequate substitution of platelets and FFP, as well as heparin reduction, requiring substitution of Factor XIII concentrate (FXIII, Fibrogammin). According to the manufacturer (CSL Behring GmbH) Fibrogammin contains 62.5 I.U./ml human FXIII with a specific activity of approximately 3.1–13.3 I.U./mg protein after standard solubilization. In comparison, a routine intramural quality analysis of 30 FFPs (male/female 16/14, age range 19–55 years, blood group distribution [%]: 0 [46.7%], A [43.3%], B [6.7%], AB [3.3%]) by the Department of Transfusion Medicine and Haemostaseology, University Hospital Erlangen-Nuremberg, Germany revealed a mean FXIII activity of 93 ± 17% (range 58–134%), with 100% equal to 1 I.U / ml. All blood components administered are listed in Table 3. Overlap of transfused blood products/volume expanders is displayed in S1 Fig. The respective Venn diagram was generated using the BioVenn online tool [21]. Administration of colloidal volume expanders, i.e. hydroxyethyl starch (HES) 6% (130/04, Fresenius Kabi Deutschland GmbH, Langenhagen, Germany), was performed in hemodynamically unstable cases secondary to intravasal volume deficiency (n = 27).

### Institutional protocol for anesthesia and cardiac surgery

Anesthesia and cardiothoracic surgery were performed according to our usual practice, as partly described previously [22]. In general, anesthesia was induced with midazolam, fentanyl and pancuronium, followed by continuous anesthesia on CPB using propofol and remifentanyl. The team of surgeons, anesthetists, perfusionists and paediatric cardiologists did not change during the study period. After midline sternotomy and heparin administration (400 I.U./kg), a bypass circuit was established with an adequately sized arterial cannula (Maquet, Hirrlingen, Germany) in the aorta and bicaval cannulation (Medtronic, Meerbusch, Germany) in the superior- and inferior venae cavae. CPB was started with a patient-adjusted cardiac index (CI) of 2.6–3.0 L/m^2^ (CI 3.0 L/m^2^ for <5kg body weight (BW), CI 2.8 L/m^2^ for 5-10kg BW, CI 2.6 L/m^2^ for >10kg BW) and cooling of patients to 28°C-32°C rectal temperature. Depending on the BW, different oxygenator models (Compactflo Evolution (EVO), D101, D100—all from Liva Nova, Munich, Germany) and CPB priming techniques were used (S1 Table). Activated clotting time (ACT) was continuously monitored aiming for values > 400 s (Hemochron Jr. Signature, International Technidyne Corporation, Edison, NJ, USA). In addition to the prime FFP had to be administered (median/range) for EVO 330ml (range 220 – 660ml), for D100 80ml (range 20-150ml) and for D101 75ml (range 20 -200ml). Furthermore surplus supplementation of washed PRBC was performed dependent on hemoglobin level to maintain a hematocrit between 25%–30% on CPB, with EVO 300ml (range 300 – 300ml), D100 100ml (range 40 – 300ml), D101 90ml (range 20 – 680ml). Myocardial protection was achieved by 30 ml/kg BW Custodiol cold crystalloid cardioplegia (Köhler Chemie, Bensheim, Germany). Weaning from CPB was supported with low-dose epinephrine (0.1 μg/kg/min) and milrinon (1.0 μg/kg/min). After CPB and routine modified ultrafiltration (MUF) [23, 24], protamine was administered in a heparin to protamine ratio of 1:1.

### Statistical analysis

The statistical analysis was performed using SAS Version 9.4 (SAS Institute Inc., Cary, NC, USA). Descriptive statistics of continuous variables are presented as mean ± standard deviation (SD) for normally distributed variables and median, minimum and maximum values for non-normally distributed variables. Categorical variables are presented as numbers and percentages. The unpaired t-test was used to compare differences between groups of chest tube drainage loss. In addition, the paired t-test was used to evaluate associations between the pre-operative and post-operative blood tests. Effects of pre-operative patient characteristics and blood values on post-operatively received transfusion, as well as on surgical parameters (RACHS-1 and Aristotle scores, duration of CPB, cross clamp time), duration of stay and on the amount of post-operative CTD were estimated using logistic regression analysis (uni-, multivariate). The effects are presented as odds ratios with 95% confidence intervals. Bivariate associations between continuous variables were analyzed using Pearson’s correlation coefficient. Statistical significance was defined as p ≤ 0.05. The minimal dataset of our study can be found in S1 Appendix.

## Results

Seventy-six patients were included in the analysis, whereof thirty patients completed a five-year follow-up at our institution with a survival rate of 96.7% (one patient deceased following re-operation). Tables 1–3 summarize the peri-operative and surgical data. There were no significant differences between the rates of female patients (n = 36, 47.4%) and male patients (n = 40, 52.6%). The majority (72.4%) of cases underwent primary cardiac surgery. Regarding body weight, 37 (48.7%) of the children were underweight (SDS < 10^th^ percentile, i.e. ≤ -1.28) at the time of surgery, with 21 (27.6%) even <3^rd^ percentile (SDS ≤ -1.88). Body weight of re-operated children remained in the same low range (Table 1). We did not observe a significant association between weight SDS and duration of CPB (data not shown).

Cyanosis was present in 26.32% of our patients pre-operatively with significant improvement (11.84%, p<0.001 McNemar test) post-operatively (S2 Table).

Our cohort encompassed CHDs listed in Table 2. Thus, a baseline adjustment of risk differences was performed using RACHS-1 [16] categories and Aristotle [17] score (Table 1), to allow comparability of the examined cases. Both RACHS-1 categories and Aristotle scores showed a positive association with the duration of CPB and cross clamp time (S2 Fig), underscoring the clinical relevance of these scores for the assessment of the severity of the surgical procedure. Median RACHS-1 category was 3 of 6 and median Aristotle score was 7.5 of 15, indicating a representative selection of cases (Table 1).

In total 67.1% (n = 51) of our patients received transfusion of FFP>PRBC>platelets, as well as PCC and fibrinogen (Table 3), with an overall repetitive transfusion (yes/no) frequency of once 22.4% (n = 17), twice 34.2% (n = 26), three times 7.9% (n = 6) and four times 2.6% (n = 2) (S1 Fig). Colloidal volume expanders (HES 6%) were substituted in 35.5% (n = 27) of cases, with a great overlap with PRBC (n = 15, S1 Fig) and FFP (n = 14, S1 Fig). We did not observe an association between the pre-operative FXIII activity and the amount of CTD loss, age or weight (data not shown).

The results of the univariate logistic regression analysis of our cohort are summarized in Table 4: Our cohort consisted of 47.4% female and 52.6% male patients, of whom 80.6% and 52.63% received blood products (PRBC, platelets, FFP, PCC, fibrinogen) after surgery, respectively, with a subsequently increased odds ratio (OR = 3.39 [1.21; 9.53]) for transfusion and female sex (Table 4). Post-operative transfusion of blood products (OR = 6.39 [1.92; 21.29]) in general and in particular the post-operative administration of PRBC (OR = 10.71 [3.64; 31.57]) and HES 6% (OR 3.00 [1.13; 7.93]) were associated with the duration of stay at PICU >48h (Table 4). Furthermore, we observed a negative association of age at operation with transfusion of blood products in general (OR = 0.85 [0.77; 0.92]) and FFP in particular (OR = 0.86 [0.79; 0.94]), following surgery (Table 4). Pre-operative body weight SDS was negatively associated with post-interventional transfusion of platelets (OR = 0.48 [0.24; 0.98], Table 4).

**Table 4:** Univariate logistic regression analysis of post-operative transfusion requirement.

Variable	Transfusion^#^ (yes n = 51 / no n = 25)	PRBC (yes n = 36 / no n = 40)	Platelets (yes n = 13 / no n = 63)	FFP (yes n = 42 / no n = 34)	HES 6% (yes n = 27 / no n = 49)
	OR (5%-95% CI)	OR (5%-95% Cl)	OR (5%-95% Cl)	OR (5%-95% Cl)	OR (5%-95% Cl)
Sex (female/male)	**3.39 (1.21–9.53)**	**1.87 (0.75–4.67)**	**1.37 (0.41–4.53)**	**1.57 (0.63–3.91)**	1.05 (0.41–2.69)
Age (LMS)	**0.85 (0.77–0.92)**	0.85 (0.77–0.93)	**0.87 (0.76–1.01)**	**0.86 (0.79–0.94)**	1.07 (0.99–1.16)
Body weight (SDS)	**0.80 (0.54–1.19)**	0.92 (0.63–1.35)	**0.48 (0.24–0.98)**	**0.80 (0.54–1.17)**	**0.86 (0.57–1.29)**
Hb (g/dl, preOP)	1.04 (0.81–1.33)	1.18 (0.93–1.51)	0.82 (0.59–1.13)	1.02 (0.81–1.29)	1.21 (0.94–1.56)
Platelet count (preOP)	1.00 (1.00–1.01)	1.00 (1.00–1.01)	1.00 (1.00–1.01)	1.00 (1.00–1.01)	1.00 (0.99–1.00)
PT (%, preOP)	1.01 (0.97–1.05)	1.01 (0.97–1.04)	0.97 (0.93–1.02)	1.00 (0.97–1.04)	1.01 (0.97–1.05)
FXIII activity (%, preOP)	1.00 (0.98–1.02)	0.99 (0.97–1.01)	0.99 (0.96–1.02)	1.00 (0.98–1.02)	0.98 (0.96–1.00)
Cyanosis (yes/no, preOP)	**6.27 (1.33–29.70)**	**7.20 (2.12–24.50)**	**2.00 (0.57–7.04)**	**1.73 (0.60–4.98)**	**2.29 (0.81–6.53)**
Stay at PICU >48h (yes/no)	**6.39 (1.92–21.29)**	**10.71 (3.64–31.57)**	**1.77 (0.53–5.90)**	**2.09 (0.82–5.35)**	**3.00 (1.13–7.93)**
Chest tube drainage loss (ml/kg)	1.07 (1.02–1.11)	1.05 (1.02–1.08)	1.00 (0.99–1.01)	1.04 (1.01–1.08)	1.00 (0.99–1.00)

^#^ includes PRBC, platelets, FFP, PCC and fibrinogen. Abbreviations: OR = estimated odds ratio, PRBC = packed red blood cells, FFP = fresh frozen plasma, HES = hydroxyethyl starch, PT = Prothrombin time ratio, ml = milliliters, kg = kilograms, h = hours, PICU = pediatric intensive care unit, ns = statistically not significant, SDS = standard deviation score, preOP = pre-operatively, LMS = LMS parameters are the median (M), the generalized coefficient of variation (S), and the power in the Box-Cox transformation (L). Blue = primary outcome, purple = secondary outcome.

RACHS-1 rank and Aristotle scores (data not shown) were only associated (OR = 1.79 [1.05; 3.07] and 1.28 [1.04; 1.59], respectively) with the post-operative decision to administer HES 6% (p<0.033 and p<0.020, respectively).

In line with the finding that both RACHS-1 and Aristotle scores reliably reflected the severity of the performed surgical procedure in our cohort (see above), we were able to determine that HES 6% was pre-dominantly administered to PICU patients (n = 27) with long CPB durations (175.2 ± 75.1 vs. 128.2 ± 57.78 minutes; p = 0.006).

The prevalence of pre-operative cyanosis was associated with the amount of post-operative transfusion in general (OR = 6.27 [1.33–29.70]) and with the decision to administer PRBC (OR = 7.20 [2.12; 24.50]) in particular, yet showed no influence on the pre-operative activity of FXIII or the post-operative CTD loss above the median (data not shown).

Interestingly, no significant associations were observed for the amount of CTD loss and pre-operative FXIII with the factors studied (Table 4). Hence, pre-operative FXIII was additionally subjected to a multivariate regression analysis (Table 5), which included CTD loss, body weight, pre-operative cyanosis and FXIII activity. Again, no significant associations were found with post-operative administration of blood products or HES 6% (Table 5). The prevalence of pre-operative cyanosis remained associated with PRBC transfusion and pre-operative body weight SDS remained negatively associated with post-interventional transfusion of platelets (Table 5, p<0.02 and p<0.05, respectively).

**Table 5:** Multivariate logistic regression analysis of post-operative transfusion requirement.

Variable	Transfusion^#^ (yes n = 51 / no n = 25)		PRBC (yes n = 36 / no n = 40)		Platelets (yes n = 13 / no n = 63)		FFP (yes n = 42 / no n = 34)		HES 6% (yes n = 27 / no n = 49)	
	OR (5%-95% Cl)	p-value	OR (5%-95% Cl)	p-value	OR (5%-95% Cl)	p-value	OR (5%-95% Cl)	p-value	OR (5%-95% Cl)	p-value
Chest tube drainage loss (ml/kg)	1.05 (1.01–1.10)	**0.03**	1.04 (1.01–1.07)	**0.02**	1.00 (0.98–1.02)	ns	1.04 (1.01–1.08)	**0.02**	1.00 (0.98–1.01)	ns
Body weight (SDS)	0.91 (0.56–1.50)	ns	1.10 (0.65–1.86)	ns	**0.46 (0.21–1.00)**	**0.047**	0.92 (0.57–1.50)	ns	0.84 (0.54–1.30)	ns
Cyanosis (yes/no, preOP)	**3.49 (0.65–18.78)**	ns	**4.87 (1.30–18.24)**	**0.02**	**2.38 (0.59–9.62)**	ns	0.90 (0.26–3.06)	ns	**2.72 (0.86–8.59)**	ns
FXIII activity (%, preOP)	1.01 (0.98–1.03)	ns	1.00 (0.97–1.02)	ns	0.99 (0.96–1.02)	ns	1.00 (0.98–1.02)	ns	0.98 (0.95–1.00)	ns

Note: The displayed p-values were not corrected for multiple comparisons. Thus, they are of exploratory nature and not meant to be interpreted in a confirmatory fashion.

^#^ includes PRBC, platelets, FFP, PCC and fibrinogen. Abbreviations: OR = estimated odds ratio, PRBC = packed red blood cells, FFP = fresh frozen plasma, PCC = prothrombin complex concentrate, HES = hydroxyethyl starch, ml = milliliters, kg = kilograms, preOP = pre-operatively, ns = statistically not significant, SDS = standard deviation score, preOP = pre-operatively. Blue = primary outcome, purple = secondary outcome.

Post-operatively, our cohort showed a significant reduction (Δ) of hemoglobin, platelet count and PT (p<0.001 for all, Table 1) and post-operative FXIII activity (p <0.039, Table 1). When comparing patients with post-operative CTD loss above the median to patients with CTD loss below the median (median CTD loss = 16.53 ml/kg), we found that patients with increased CTD loss in fact had significantly higher FXIII activity levels (Table 6, p<0.002), while no such difference was found for hemoglobin, platelet count or PT (Table 6).

**Table 6:** Comparison of post-operative blood parameters of patients with chest tube drainage loss below/above the median.

	< Median CTD loss (ml/kg)*	> Median CTD loss (ml/kg)*	p-value**
ΔHb (pre- vs. postOP)	0.839 ± 0.170	0.893 ± 0.163	ns
	n = 37	n = 35	
ΔThrombocyte count (pre- vs. postOP)	0.796 ± 0.359	0.822 ± 0.268	ns
	n = 37	n = 35	
ΔPT (pre- vs. postOP)	0.895 ± 0.429	0.936 ± 0.317	ns
	n = 37	n = 32	
ΔFXIII (pre- vs. postOP)	0.921 ± 0.290	1.126 ± 0.244	**0.0024**
	n = 35	n = 33	

* median CTD loss = 16.53 ml/kg.

** = unpaired two-sided Student’s t-test.

Legend: Δ = Difference of pre- to post-operative values in percent decimal; preOP = pre-operatively, postOP = post-operatively, vs. = versus, PT = Prothrombin time ratio, CTD = chest tube drainage.

Interestingly, higher post-operative FXIII activity was correlated (Pearson r = 0.24, p = 0.033) with a prolonged PICU stay >48 h. Concomitantly, patients with post-operative CTD loss above the median had received significantly more intra-operative transfusion in total (i.e. priming and individually added) of both PRBC and FFP (Table 7, p<0.001).

**Table 7:** Comparison of intra-operative volume management and oxygenator models.

mean ± SD	Descriptive Analysis			Statistical Analysis		
	EVO (n = 23)	D100 (n = 15)	D101 (n = 37)	< Median CTD loss* (ml/kg)* (n = 37)	> Median CTD loss* (ml/kg)* (n = 38)	p-value
Jonosteril^1^ (ml/kg)	23.57 ± 11.54	18.00 ± 7.08	26.57 ± 17.22	24.38 ± 12.72	23.51 ± 15.82	ns
PRBC (ml/kg)	1.45 ± 4.26	55.80 ± 13.32	25.85 ± 21.39	11.97 ± 21.94	36.41 ± 21.93	**<0.0001**
FFP (ml/kg)	2.05 ± 3.81	29.42 ± 14.21	12.88 ± 12.26	5.32 ± 9.46	20.22 ± 14.68	**<0.0001**

Descriptive analysis of the total amount of prime and individually administered blood products and Jonosteril categorized in groups depending on the oxygenator model (i.e. EVO, D100, D101) or chest tube drainage (CTD) loss above the median. For the latter, a statistical analysis was performed;

* median CTD loss = 16.53 ml/kg;

^1^ Fresenius Kabi Deutschland GmbH, Langenhagen, Germany.

The total amount of intra-operatively used PRBC (ml/kg) and FFP (ml/kg) correlated with the Aristotle score (r = 0.36, p<0.002 and r = 0.29, p<0.011, respectively). No correlation was found for intra-operative Jonosteril.

**Fig 2:**
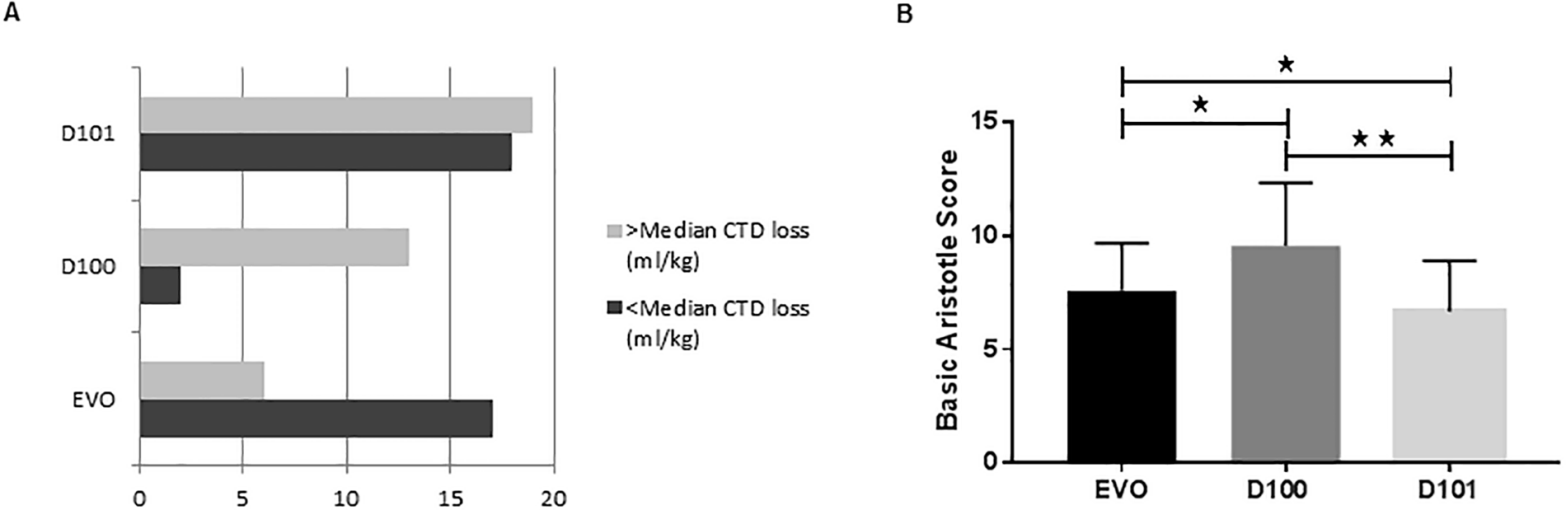
A) Displayed is the number of patients (x-axis) per oxygenator model (y-axis), grouped according to the amount of CTD loss above (bright) or below (dark) the median. B) Comparison of the distribution of the complexity of cases (Aristotle score) among oxygenator models. Patients oxygenized with the D100 model had significantly higher Aristotle scores of 9.54 ± 2.76 (mean ± SD) when compared to patients on EVO and D101 (p<0.05 and p = 0.004, respectively). The mean Aristotle score of patients on EVO was 7.58 ± 2.09, while patients on D101 had the lowest scores 6.66 ± 2.23 (p<0.029 vs. EVO; p<0.004 vs. D100). Patients oxygenated on the D100 had the lowest BW with a median of 3.5 kg (range 2.3–4.6 kg) (see S1 Table). * = p<0.05, ** = p<0.004.

Further, post-operative CTD loss in our cohort was apparently associated with the choice of oxygenator models (S1 Table, Fig 2A). The number of patients with CTD loss above the median was higher following oxygenation with D100, while lower when EVO system was used (Fig 2A). However, this difference is largely explained by the severity of cases (Fig 2B) and their weight-based (S1 Table) distribution among the three oxygenator models used. There was heterogeneity in CPB priming. While the majority of patients received FFP and PRBC (D100/101 oxygenators), 27.6% (i.e. older patients on EVO oxygenators) did not (S1 Table). However, in these cases FFP and PRBC were individually introduced in 28.6% and 14.3% during the course of CPB, respectively, and the ΔFXIII activity (% of pre-operative value) of EVO 100.3 ± 31.0% (mean ± SD) was comparable to activity levels found in patients on D100 (119.4 ± 38.8%) and D101 (96.5 ± 19.5%) systems.

The analysis of the associations between intra-operative volume management (i.e. total priming and transfusion) and post-operative blood tests with post-operative transfusion requirements is displayed in Table 8. The decision to administer blood products after surgery seemed independent from the total amount (ml/kg) of Jonosteril, PRBC and FFP used for CPB (Table 8). Univariate analysis of intraoperative priming and post-operative alterations (Δ, pre- vs. post-operatively) of Hb, platelet count, PT and especially FXIII activity showed no association with post-operative transfusion requirements (Table 8).

**Table 8:** Analysis of the associations between intra-operative volume management and post-operative blood tests / transfusion requirements.

	Variable	Transfusion^#^ (yes n = 51 / no n = 25)	PRBC (yes n = 36 / no n = 40)	Platelets (yes n = 13 / no n = 63)	FFP (yes n = 42 / no n = 34)	HES 6% (yes n = 27 / no n = 49)
		OR (5%-95% CI)	OR (5%-95% Cl)	OR (5%-95% Cl)	OR (5%-95% Cl)	OR (5%-95% Cl)
**Intra OP**	Jonosteril^1^ (ml/kg)	0.99 (0.96–1.02)	0.98 (0.94–1.01)	1.00 (0.96–1.05)	0.99 (0.96–1.03)	0.97 (0.94–1.02)
	PRBC (ml/kg)	1.05 (1.02–1.08)	1.04 (1.02–1.07)	1.02 (0.99–1.04)	1.03 (1.01–1.06)	1.00 (0.98–1.02)
	FFP (ml/kg)	1.08 (1.02–1.14)	1.07 (1.03–1.11)	1.03 (0.99–1.07)	1.05 (1.01–1.09)	1.02 (0.99–1.05)
**Post OP**	ΔHb (%, pre- vs. postOP)	1.02 (0.99–1.06)	0.99 (0.97–1.02)	1.02 (0.98–1.05)	1.01 (0.98–1.04)	0.99 (0.96–1.02)
	ΔPlatelet count (%, pre- vs. postOP)	1.01 (0.99–1.03)	1.01 (1.00–1.03)	0.97 (0.94–1.00)	1.01 (0.99–1.03)	1.01 (0.99–1.02)
	ΔPT (%, pre- vs. postOP)	1.00 (0.99–1.02)	1.00 (0.99–1.02)	0.99 (0.96–1.01)	1.00 (0.99–1.02)	1.01 (0.99–1.02)
	ΔFXIII activity (%, pre- vs. postOP)	1.01 (0.99–1.03)	1.03 (1.01–1.05)	1.00 (0.98–1.03)	1.01 (0.99–1.02)	1.02 (1.00–1.05)

^#^ includes PRBC, platelets, FFP, PCC and fibrinogen. Abbreviations: OR = estimated odds ratio, PRBC = packed red blood cells, FFP = fresh frozen plasma, PT = Prothrombin time ratio, HES = hydroxyethyl starch, ml = milliliters, kg = kilograms, IntraOP = intra-operatively, PostOP = post-operatively, vs. = versus.

^1^ Fresenius Kabi Deutschland GmbH, Langenhagen, Germany. Blue = primary outcome.

## Discussion

We analyzed whether pre- and post-operative FXIII levels are associated with the requirement of blood product supplementation and CTD loss in the first 48 h following open heart surgery for infants with CHD involving CPB. We found that post-operative transfusion requirements and administration of HES 6% seemed independent of both pre- and post-operative FXIII activity and CTD loss in our cohort.

Our findings are in line with previous studies, including our own [13], that provided no evidence of a significant correlation between FXIII activity and CTD loss or any advantage of a routine factor XIII supplementation [3–5, 12, 13]. In detail, we were previously able to show that a single post-operative administration of FXIII reduced effusions after cardiac surgery for the first 24 h, but not thereafter [13]. Chandler et al. [5] also observed a time-limited correlation between FXIII activity and post-operative CTD loss during the first 2 h after PICU admission only. Similarly, FXIII substitution in adults had no significant influence on CTD loss or transfusion requirements, either [3, 4].

Based on published reports for adult [4, 25] and pediatric [26, 27] cohorts after cardiac surgery we expected a post-operative drop of FXIII between 30–70%, secondary to hemodilution. We were surprised to find that post-operative FXIII activity in our cohort was unchanged and rather elevated in patients with CTD loss above the median. Concomitantly, higher levels of FXIII after surgery correlated with an increased duration of stay in our PICU. In line with our findings, a recent analysis of forty-four comparably smaller children (age < 1 year, weight < 10 kg) by Gertler et al. [12] showed, that acquired FXIII deficiency did not develop in the context of a reconstituted blood prime with adult FFP and PRBC, with stable FXIII activity throughout the entire peri-operative course, in contrast to all other post-operative coagulation tests. Thus, Gertler et al. [12] concluded that FXIII supplementation is unnecessary for the majority of pediatric cardiac surgical patients. The benefit of using FFP for priming has been discussed in detail by others: Its use for priming is advantageous over the mere post-operative administration [28] and significantly reduces transfusion rates and donor exposures [29, 30]. The majority of our patients received reconstituted blood priming for CPB or administration of FFP and PRBC while being on CPB.

Gertler et al. [12] surveyed RACHS-1 ranks and Aristotle score but disregarded them as variables in their analysis of FXIII, CTD loss and the need for post-operative transfusion. Interestingly, despite a significant correlation of RACHS-1 and Aristotle with the duration of CPB and cross clamp time in our cohort, we observed a positive association with the post-operative decision to administer HES 6% only. This finding could indicate an increased capillary leakage associated with a longer duration of CPB, as shown by others [31]. When analyzing the association of HES 6% and blood product administration with the duration of stay, we were not surprised to find that patients in need for transfusion had to be treated longer on PICU than others. Strikingly, however, duration of stay at our PICU was not associated with any complexity-indicator (RACHS-1, Aristotle, CPB duration, cross clamp times) of the surgical procedure.

This finding is in contrast to other studies [32, 33]. Noteworthy, however, RACHS-1 category seemed to explain only <20.0% of both the total and individual post-operative lengths of hospital stay in the latter study [33]. It needs to be pointed out, that while RACHS allows comparison among groups of patients, it is not suited for prediction of individual outcomes in high-risk patients [16]. Our results might have been affected by confounding variables, as discussed by Simsic et al. [34]. In their single-center series of high-risk newborns, risk adjustment for congenital heart surgery did not sufficiently predict outcome variables and in-hospital mortality [34]. These confounders included instable hemodynamics, type of cardiac defect, palliation versus complete repair and especially weight [34], as small for gestational age (SGA) infants with CHD are a priori prone to suffer from post-operative complications with an increased mortality compared to their appropriate for gestational age (AGA) counterparts [35, 36].

The OR for a post-operative PICU stay >48 h were 10.71 [3.64; 31.57] for PRBC and 3.00 [1.13; 7.93] for HES 6%, with an overall OR of 6.39 for transfusion of blood products in general. This finding might underline the importance for red-cell transfusion avoiding strategies, as just recently proclaimed by Lacroix et al. for the treatment of anemia in critically ill children [37]. While transfusion of PRBC is a common practice to optimize tissue oxygenation, especially in patients with shock, it is assumed that it might exert adverse effects via immunomodulation and the accumulation of inflammatory mediators via storage-based product alterations [38]. Similar effects are observed following administration of FFP [39]. In this context it is noteworthy that we observed a negative association of age with the post-operative transfusion of blood products in general (OR 0.85 [0.77; 0.92]) and FFP in particular (OR 0.86 [0.79; 0.94), which might indicative of a more restrictive transfusion strategy in our older children. However, it has to be noted that there might have been other relevant factors involved in the duration of PICU stay [40–44] in our cohort, such as the duration of mechanical ventilation, delayed or secondary sternal closure and infection, which were not included in our analysis.

Historically, neonatal PRBC transfusions are regularly used in cyanotic CHD to increase hemoglobin with a potential increase in oxygen carrying capacity. Following our intramural standard, PRBC were administered at a hemoglobin (Hb) level of 14 g/dl in cyanotic patients and 10 g/dl in non-cyanotic patients. Thus, the finding of a positive association of cyanosis and PRBC transfusion in our study (OR = 4.87 [1.30; 18.24]) seems comprehensible. However, this regimen is now being critically discussed, as PRBC transfusions carry a number of associated risks that may be translated into increased patient morbidity and mortality [45, 46]. Unfortunately, sufficient evidence to assess the impact of PRBC transfusion on patients with CHD undergoing cardiac surgery is currently lacking [46].

The post-hoc finding that our female pediatric patients were prone (OR = 3.39 [1.21; 9.53]) to receive transfusions post-operatively was startling. It has been shown that female adults undergoing surgery are significantly more transfused than men [47–49], potentially due to their increased bleeding tendency [49, 50]. Following surgery, these women have an increased risk for adverse outcomes and death, which can be attributed to higher allogeneic transfusion rates to some extent [47, 49]. In fact, a recent study [49] involving adults undergoing cardio- and orthopedic surgery revealed higher transfusion rates and volumes in women when compared to men, independent of the type of surgery. This outcome resulted from a uniform application of absolute transfusion thresholds irrespective of a patient’s sex. Noteworthy, uniform transfusion triggers are applied in routine clinical practice (including our own during the time of study) disregarding sex [49, 51], with adult transfusion guidelines concomitantly focused on absolute haemoglobin values [52–55].

Low body weight has been as shown to adversely affect newborn surgical mortality [56–58]. The auxologic analysis of our cohort revealed that 48.7% of patients had a body weight below the 10^th^ percentile. However, our study incorporated all age groups. Thus, low body weight is rather indicative of failure to thrive in our patients, which argues for the need of nutritional counseling of patients with CHD [59].

The finding that body weight was inversely associated with the transfusion of platelets (multivariate OR = 0.46 [0.21; 1.00]) in our cohort might reflect our institutional transfusion protocol at the time of study, with a transfusion nadir of 50x10^3^/μl, independent of age. The combination of thrombocytopenia and platelet dysfunction in neonates has been discussed to contribute to a higher incidence of bleeding (reviewed by [60]). However, recent studies seem to indicate a poor correlation between the severity of thrombocytopenia and clinically significant bleeding, calling upon an improvement of the assessment of primary hemostasis and bleeding risk in neonates (reviewed by [60]).

Using an approach similar to our study, Bocsi et al. [61] aimed to predict effusions following CPB surgery in pediatric patients via the pre-operative determination of serologic indicators of inflammation (such as e.g. factors of the complement system). Interestingly, they found that prodromal differences in the immune system and capillary permeability status might be relevant for an overshooting immune response, putting children at risk for post-operative effusions and capillary leak syndrome [61]. The simultaneous activation and interaction of inflammatory and coagulation processes succeeding injury is an ancient, phylogenetic adaptive response and helps protecting the organism from both infection and blood loss [1, 62].

## Conclusion

Taken together, our results show that post-operative transfusion requirements and administration of HES 6% seemed independent of both pre- and post-operative FXIII activity and CTD loss in our cohort. It remains to be determined, whether the use of reconstituted blood prime with adult FFP for CPB priming sufficiently averted the acquisition of FXIII deficiency in our study. As a consequence, routine administration of FXIII might have been unnecessary in the vast majority of patients to reduce transfusion requirements post-operatively. Nevertheless, in certain cases with low FXIII and severe CTD-loss, FXIII supplementation should still be considered a therapeutic option. Especially the participation of FXIII in the immunologic cross-talk could indicate further implications that warrant future elucidation.

### Limitations

Our retrospective study is limited by the fact, that HES 6% was used as a post-operative volume expander. As the administration of HES has been associated with negative outcomes, especially renal failure and reduced hemostasis [63–65], this therapeutic approach is no longer practicable. Thus, while our results regarding FXIII are similar to a comparable HES-free study [12], their predictive value needs further validation in other patients, not least because of certain cohort inhomogeneity: We did not limit our study to the analysis of only a certain subset of CHD, which could have potentially masked cardio-surgery specific effects on CTD loss and transfusion requirements The heterogenic use of reconstituted blood prime, especially in older children, and the subsequent choice of oxygenator models was strongly influenced by patient auxology and age, as discussed above. The fact that our cohort consisted of children and adolescents before and after the onset of puberty could have negatively influenced the identification of sex-specific findings and their interpretation, such as the pronounced post-operative transfusion of female patients. Additionally, prematurity and genetic disorders, as major causes for low body weight [59, 66], were not included as variables in our analysis. Furthermore, pre-operative inflammatory parameters, as described by Bocsi et al. [61], were not evaluated in parallel to FXIII in our study. Analysis of these factors should be addressed in future studies, as FXIII is known to influence infection control [1, 67] via interaction with complement factors and inflammatory cells.

Further limitations are the retrospective study design, which might have resulted in a selection bias (e.g., regarding age, priming, type of operation), and the relatively small cohort size, which might have resulted in a reduced statistical power of the performed hypothesis tests. While the confidence intervals reported here provide at least some information about the power of the analyses, this issue could be addressed more properly by a prospective study design with a priori sample size and power calculations. We also emphasize that most of the reported p-values (e.g., in Table 5) must be interpreted in a non-confirmatory (exploratory) fashion. Based on the amount of tests and the limited sample size, it was infeasible to implement a correction procedure for multiple testing enforcing strict family-wise error rate control.

## Supporting information

S1 Table Priming. ^#^ Livanova Munich, Germany; ^1^ Fresenius Kabi Deutschland GmbH, Langenhagen, Germany; ^2^ Ratiopharm GmBH, Ulm, Germany; ^3^ Roche, Grenzach- Whylen, Germany; ^4^ Verla-Pharm, Tutzing, Germany; ^5^ Serag-Wiessner, Naila, Germany; ^6^ Rotexmedica, Trittau, Germany; ^7^ CSL Behring, Hattersheim am Main, Germany; ^8^ prepared by the pharmacy of the university hospital Erlangen, Germany.
(XLSX)

S2 Table Overview of pre- and postoperative prevalence of cyanosis. # includes PRBC, platelets, FFP, PCC and fibrinogen. Abbreviations: PRBC = packed red blood cells, FFP = fresh frozen plasma, HES = hydroxyethyl starch, preOP = pre-operatively, postop = post-operatively.
(XLSX)

S1 Fig Venn-diagram illustrating the overlap of post-operatively administered blood products and volume expanders. (TIF)

S2 Fig Pearson correlation of RACHS and Aristotle scores with cross clamp time and duration of cardiopulmonary bypass (CPB). (TIF)

S1 Appendix Minimal dataset. (XLSX)
