# Supplementary figures and images for: Influence of factor XIII activity on post-operative transfusion in congenital cardiac surgery—A retrospective analysis
Source: PLoS One. 2018 Jul 10;13(7):e0199240. doi: 10.1371/journal.pone.0199240 (PMC6038983; doi:10.1371/journal.pone.0199240)

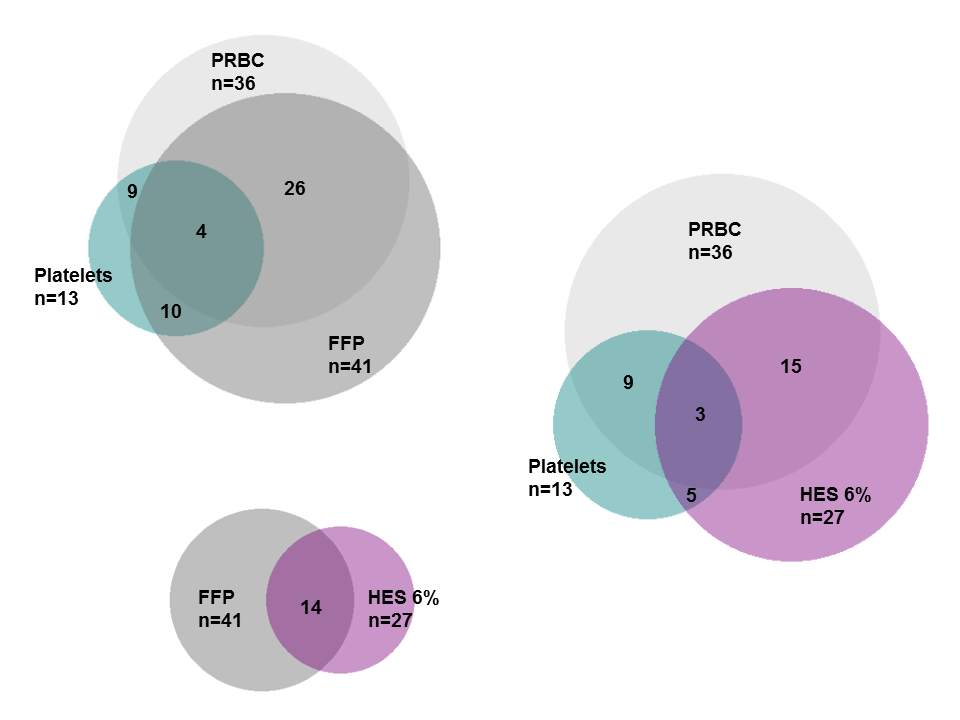

Supplement: S1 Fig — (TIF) [file pone.0199240.s003.tif]

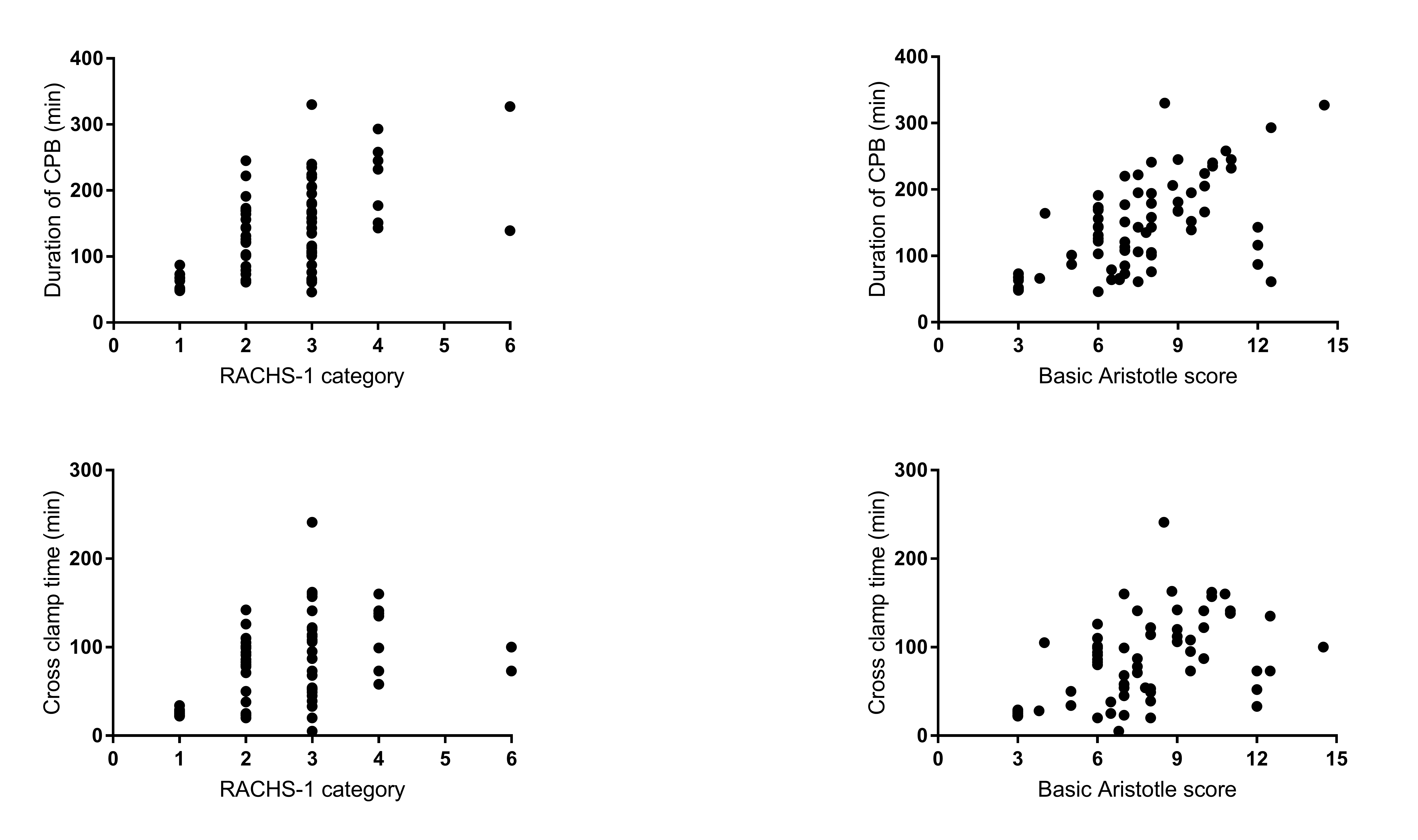

Supplement: S2 Fig — (TIF) [file pone.0199240.s004.tif]
